# Prosthetic Dentistry

**Published:** 1881-08

**Authors:** E. L. Kellogg


### PROSTHETIC DENTISTRY.

By E. L. Kellogg, D. D. S.

Dentistry has been known and practiced from the earliest ages. It was for a long time a part of the physician's duty, as it is yet in some localities. The principal attention then paid to it was the extraction and sometimes the replacement of the lost teeth, with an artificial substitute, made in a rude manner. But little attention was then given to the prevention of diseases of the mouth and teeth. History tells us the Egyptians and the Greeks were the first people who gave any attention to this subject. Of course their knowledge must have been quite limited, and their mode of operation accordingly crude. Yet, to-day, operations are performed, artificial substitutes are made, which would not compare favorably with the work of the ancient workman with his chisel and block of ivory. It is to be presumed that the first operation was the extraction of the teeth for the relief of pain. Though we have no account of the operation as conducted in those days, it is very probable that the traditional hammer, chisel and blacksmith's tongs came into use for this purpose, followed by the various forms of keys, forceps, etc. Though never a pleasant operation, it must have been approached with much dread by the patient in those days. The loss of these organs would naturally suggest the idea of replacing them with some substitute, both serviceable for purposes of mastication and bearing some resemblance to the natural teeth. This must have been quite a perplexing problem in those days, as now, with the perfected materials of the present day it requires much skill and experience, and is then sometimes quite difficult to supply a satisfactory substitute. After the preparation of the mouth, the operator of to-day takes the impression with one of the many materials in use; but the operator in those days used to adapt his material to the gums by constant observation of the parts and by carving the ivory and bone so as to fit as well as possible, the gums. This was necessarily a very imperfect operation and required great skill on the part of the operator, accompanied with more determination by the patient than is usually exhibited now-a-days. The teeth were at first carved out of the original block with the plate. This method was in use for a long time, and the teeth thus made are said to have been hard to detect from the natural organs. Later than this the teeth were attached by ligatures and wires. A very common method of replacement was by pivoting. This method could only be used in cases of single-rooted teeth. Both wooden and wire pivots were used. Among the materials then used were ivory, teeth of cattle, sheep and human teeth. These methods seem to us now very crude, but no doubt they were as well, if not better appreciated than the work of the dentist is to-day. Having briefly reviewed some of the methods of the past; I will now consider the work of to-day. Since the loss of the teeth affects the speech, mastication and the appearance of the individual so much, it becomes a matter of the greatest importance to supply the loss. The extraction of the teeth should first be carefully performed. Next the alveolar ridge should be carefully examined, and if too long or too thick, the edges of the process should be removed so that in adapting artificial teeth too much prominence can be avoided. A suitable wash should be given the patient to keep the mouth pure and to facilitate the healing of the gums. For this purpose "Phenol Sodique" reduced three-fourths is an excellent preparation, and seems to give general satisfaction. It is best that the patient should wait till thorough absorption of the bone or process has taken place, before wearing a substitute.

Though anticipating my subject a little, I would say a word about so-called temporary plates. The majority of patients who come to the dentist for artificial teeth, wish to have a temporary set of teeth inserted shortly after extraction. They come with the idea that a set of teeth can be inserted at once, after extraction. Many have never heard any reason why they should not wear them at once, and others assume that they know all about it and will not accept any advice the dentist may offer. This first class of people may be induced to wait the proper time, viz: from four to six months after extraction. The dentist should endeavor to dissuade his patients from wearing temporary sets as they cause irritation, prevent healthy absorption from going on, and by sucking the gum close to the plate, cause a loose and flabby condition of this surface. Without the plate the gums will ordinarily heal firmly and evenly, making a good solid ridge which will firmly sustain a substitute. The advocates of these plates claim that they keep the muscles of the face out in a more natural position, but as these can be restored by a proper adaptation of the permanent set, it is a matter of minor importance when compared with a good firm gum as a foundation for the permanent substitute.

Wax was first used for taking impressions. Gutta percha, plaster and various compositions have been used for this purpose, and each has its advocates. I believe wax and plaster are the two most in use now. Wax is chiefly used in taking partial impressions of either jaw, and as a matrix for plaster in impressions of the lower jaw when all the teeth are absent.

It is a good plan to rinse the mouth with tepid water just before taking the impression, as this cleans away the mucus, leaving the mouth in better condition. In heating the wax it should be well and evenly heated, and it is considered best to use dry heat, a gas jet or flame of lamp. It may be prepared quickly by heating in warm water, care being taken in either case not to work it when too hot, and in the latter case wipe the wax dry before using. Having heated the wax and placed it in the cup, care should be taken in getting the impression, not to carry the cup too far back in the mouth. In the upper jaw the cup should be carried to place with an upward and forward movement. When ready to remove, lift the lip, admitting a little air, and the cup may be removed slowly and easily. In using plaster the method is much the same, only care must be taken to have the patient lean forward immediately after inserting the cup, so that no loose pieces of plaster may fall into the throat. See that the loose tissue is drawn away, and do not press hard when carrying to place. The finer the plaster the more uniform will be its crystalization. It should be mixed in warm water with a little salt or sulphate of potash thrown in which makes the plaster set quicker and renders the operation shorter, and less disagreeable to the patient. The bite should next be obtained to get a correct articulation. Having taken the impression the next step is to make a model. The surface of the impression should be carefully coated with collodion not getting too much upon it, and then the plaster mixed to the consistency of cream should be poured into it. If a metal plate is to be swaged the model should be made at least three inches deep. It need not be made so deep for a rubber plate. It should always be thick enough so it will not warp, however. When thought sufficiently hard, the impression may be cut away from the model, for, if left too long in the impression, it will result in bulging of the palatine arch and make a "rocking-plate." When a plate is to be swaged the ridge in the impression should be deepened, being careful to retain the general outline. The plaster cast should be trimmed neatly and smoothly and covered with a coating of shellac or some varnish. A groove should be cut where the heel of the plate comes shallow over the median line or hard parts, and shallow over the soft parts, so that the plates may set well up against the soft gums. When the undercut in front is deep the heel of the cast should project so it may slip easily from the sand in molding. The undercut may often be trimmed out of the metal die.

After the counter die is made, care should be taken in swaging the plate that it does not buckle and crack, and the die should not be struck too hard or long as the plate may be spread thereby. A good plan, after the plate is swaged up is to try it in the mouth, and while you can have the patient's presence, adapt the plate thoroughly to the gums by the use of the pliers. A better fit may often be thus made. When the plate fits perfectly it is ready for the adaptation and fitting the teeth. As to bases, various metals have been used. It is not known when metals first came into use for this purpose. Gold was probably the first metal used, and still holds its place in the front rank as the best metal for this purpose. As it is so expensive, platinum, silver, aluminium, etc., have been used. An alloy of platinum was once used, but now forms the base in continuous gum-work, and the pins in porcelain teeth. Silver oxidizes very rapidly in the mouth, but is used considerably. Aluminium as a base was not at first satisfactory. No solder has as yet been found whereby teeth can be secured to this base. The plan of attaching the teeth with rubber makes a neat plate. Another method of attachment by flowing a composition metal about the pins of the teeth and into sockets in the plate makes an attachment, thought by many superior to rubber. Tin has been used as a base, but has a disagreeable taste. An alloy of tin, silver and bismuth, called cheoplasty, was for a while received favorably, but was soon abandoned. Continuous gum-work gives to the dentist the best opportunity to meet all requirements of color of gums and arrangement of the teeth. I believe some writers claim more for it than it really deserves. It certainly makes, in the majority of cases, the finest looking denture that can be made. Vulcanite caused a great change in the manufacture of artificial dentures. It is very easily manipulated, and consequently very much used. Probably a great many of the dentists using this material have never made a metal base. There has been much said of salivation being caused by the wearing of rubber plates, but I believe it has been generally conceded that rubber does not often have this injurious effect upon the system when healthy. It does, however, have a bad effect by preventing the passage of impressions of heat and cold to the gums. It irritates the gums of some persons very much, and helps to bring about an ulcerated condition. Of course, there are good and poor qualities of this material. I have seen plates of the red rubber where the color could be rubbed off upon the brush-wheel. Such a plate must be injurious. It is quite useful in cases of cleft-palate—of which more hereafter. Celluloid made of cellulose and gum-camphor was soon introduced after rubber. It is now much better received by the profession than at first, and is useful in many ways. It possesses the desirable quality of taking a color, closely resembling the healthy gum. In a late number of the "Cosmos" is described a plate made of rubber and celluloid combined, which would seem, from the description, to be a good plate—not only are these metals and gums used to supply deficiencies in the arches of teeth, but they are also used to restore lost parts, such as the palate or nose. These are ancient inventions and were in use many years ago. They are now much improved, and are great helps to those so unfortunate as to need these artificial substitutes. The dentist will meet with cases where there is a simple hole through the palatal bone, caused by disease, or he may meet with cases where the whole palate is cleft. A surgical operation is often effective, but there are many cases where the obturator is the only remedy. For a simple defect in the bone a plate used to be made with a sponge attached to its palatal surface to fill the opening. A raised piece of metal attached to the palatal surface of the plate fills the defect very effectually. A velum of soft rubber may be attached to the metal or hard rubber plate. Where no teeth are wanting, the plate may be attached to teeth upon opposite sides of the mouth, with clasps thus being held in place. Atmospheric pressure is also used. The obturator may be applied with either full or partial dentures. Regulating the teeth comes in the work of the prosthetic dentist. Metal and rubber plates are both used for this purpose, and help in a great measure to reform the evil effects of contracted jaws and neglected deciduous teeth. There is not room here to discuss the different modes of regulation. Plates are used with rubber bands and without them, and also appliances made of metal whose regulating power lies in springs or nuts and bolts intelligently applied. These often obviate any necessity for extraction of teeth to cure irregularity. Beside artificial palates we have artificial noses, parts of the lips and gums supplied by the ingenious fitting of a platinum or other plate to the lost part. Shaping it so as to resemble the part to be supplied and covering it with enamel colored so as to closely resemble the surrounding skin and tissues. These, though they may be inconvenient to wear, still must give great satisfaction to the afflicted, both in assisting speech and mastication, as well as in the improved appearance. To replace such prominent teatures well, requires great skill, and is not accomplished by the ordinary practitioner.

Whatever base may be used, considerable skill is requisite in the arrangement of the teeth so as to give satisfaction to the wearer and appear natural to the casual observer. Some claim that there is no satisfaction in Prosthetic Dentistry, but I think that idea wrong. There are, certainly, many mouths presented in which it is impossible to put a neat, well-appearing and serviceable plate, but on the contrary, I think the majority of mouths may be well fitted and with a degree of satisfaction to both the patient and the dentist. This success has undoubtedly been largely due to the state of perfection to which the manufacture of mineral teeth has been brought. When these teeth were first introduced in this country, in 1817, they were thought quite an improvement. But look at them then, with the appearance of a split bean and not in the least translucent. They, however, could not decay, nor become offensive in such manner. They were at first plain, having no gums. Dr. S. S. Stockton was the first person to manufacture them for the profession. They were made to be fastened to the plate by wires and ligatures. After a time, the present mode of fastening by pins was devised, and perhaps no better attachment can be made. S. S. White, has made many improvements in the manufacture of these teeth since the year 1844. At his death, a little over a year ago, he had one of the largest manufactories in the world.

Justi makes excellent teeth now, and there is progress in this direction yearly. Now I have given a brief review of this subject as far as I find it possible. It can not be well condensed in so few pages, but I trust it may convey the idea of the subject. This department of dentistry demands the attention and encouragement of the profession.

Though the laboratory may not be so pleasant and agreeable as the operating room, yet it demands as much or more attention, and much of the dentist's reputation depends upon his work there performed. Though there are many discouragements, yet it is to be hoped we may all strive to do justice to this branch of our profession.

